# Supplementary material for: A fault-intrusion-tolerant system and deadline-aware algorithm for scheduling scientific workflow in the cloud
Source: PeerJ Comput Sci. 2021 Nov 2;7:e747. doi: 10.7717/peerj-cs.747 (PMC8576554; doi:10.7717/peerj-cs.747)
Supplement: Supplemental Information 6 [file peerj-cs-07-747-s006.zip › Myproject1.1/docs/index-files/index-21.html]

V-Index


JavaScript is disabled on your browser.


- Overview
- Package
- Class
- Use
- Tree
- Deprecated
- Index
- Help

- Prev Letter
- Next Letter

- Frames
- No Frames

- All Classes

A B C D E F G H I J L M N O P Q R S T U V W Z 


## V

valueOf(String) - Static method in enum org.workflowsim.utils.ClusteringParameters.ClusteringMethod
:   Returns the enum constant of this type with the specified name.

valueOf(String) - Static method in enum org.workflowsim.utils.Parameters.FTCFailure
:   Returns the enum constant of this type with the specified name.

valueOf(String) - Static method in enum org.workflowsim.utils.Parameters.FTCMethod
:   Returns the enum constant of this type with the specified name.

valueOf(String) - Static method in enum org.workflowsim.utils.Parameters.FTCMonitor
:   Returns the enum constant of this type with the specified name.

valueOf(String) - Static method in enum org.workflowsim.utils.Parameters.PLNMethod
:   Returns the enum constant of this type with the specified name.

valueOf(String) - Static method in enum org.workflowsim.utils.Parameters.SCHMethod
:   Returns the enum constant of this type with the specified name.

valueOf(String) - Static method in enum org.workflowsim.utils.ReplicaCatalog.FileSystem
:   Returns the enum constant of this type with the specified name.

values() - Static method in enum org.workflowsim.utils.ClusteringParameters.ClusteringMethod
:   Returns an array containing the constants of this enum type, in
    the order they are declared.

values() - Static method in enum org.workflowsim.utils.Parameters.FTCFailure
:   Returns an array containing the constants of this enum type, in
    the order they are declared.

values() - Static method in enum org.workflowsim.utils.Parameters.FTCMethod
:   Returns an array containing the constants of this enum type, in
    the order they are declared.

values() - Static method in enum org.workflowsim.utils.Parameters.FTCMonitor
:   Returns an array containing the constants of this enum type, in
    the order they are declared.

values() - Static method in enum org.workflowsim.utils.Parameters.PLNMethod
:   Returns an array containing the constants of this enum type, in
    the order they are declared.

values() - Static method in enum org.workflowsim.utils.Parameters.SCHMethod
:   Returns an array containing the constants of this enum type, in
    the order they are declared.

values() - Static method in enum org.workflowsim.utils.ReplicaCatalog.FileSystem
:   Returns an array containing the constants of this enum type, in
    the order they are declared.

variance(List<Double>) - Static method in class org.cloudbus.cloudsim.util.MathUtil
:   Variance.

VerticalBalancing - Class in org.workflowsim.clustering.balancing.methods
:   VerticalBalancing is the same vc as in org.workflowsim.clustering.

VerticalBalancing(Map, Map, int) - Constructor for class org.workflowsim.clustering.balancing.methods.VerticalBalancing
:   Initialize a VerticalBalancing object

VerticalClustering - Class in org.workflowsim.clustering
:   VerticalClustering merges tasks at the same pipeline

VerticalClustering(int) - Constructor for class org.workflowsim.clustering.VerticalClustering
:   Initialize a VeriticalClustering object

Vm - Class in org.cloudbus.cloudsim
:   Vm represents a VM: it runs inside a Host, sharing hostList with other VMs.

Vm(int, int, double, int, int, long, long, String, CloudletScheduler) - Constructor for class org.cloudbus.cloudsim.Vm
:   Creates a new VMCharacteristics object.

vm2record - Static variable in class org.workflowsim.failure.FailureMonitor
:   VM ID to a Failure Record.

VM\_BROKER\_EVENT - Static variable in class org.cloudbus.cloudsim.core.CloudSimTags
:   Denotes an internal event generated in a Broker

VM\_BW - Static variable in class org.cloudbus.cloudsim.examples.power.Constants


VM\_CREATE - Static variable in class org.cloudbus.cloudsim.core.CloudSimTags
:   Denotes a request to create a new VM in a Datacentre With acknowledgement information sent by
    the Datacentre

VM\_CREATE\_ACK - Static variable in class org.cloudbus.cloudsim.core.CloudSimTags
:   Denotes a request to create a new VM in a Datacentre With acknowledgement information sent by
    the Datacentre

VM\_DATA\_ADD - Static variable in class org.cloudbus.cloudsim.core.CloudSimTags
:   Denotes an event to send a file from a user to a datacenter

VM\_DATA\_ADD\_ACK - Static variable in class org.cloudbus.cloudsim.core.CloudSimTags
:   Denotes an event to send a file from a user to a datacenter

VM\_DATA\_DEL - Static variable in class org.cloudbus.cloudsim.core.CloudSimTags
:   Denotes an event to remove a file from a datacenter

VM\_DATA\_DEL\_ACK - Static variable in class org.cloudbus.cloudsim.core.CloudSimTags
:   Denotes an event to remove a file from a datacenter

VM\_DATACENTER\_EVENT - Static variable in class org.cloudbus.cloudsim.core.CloudSimTags
:   Denotes an internal event generated in a PowerDatacenter

VM\_DESTROY - Static variable in class org.cloudbus.cloudsim.core.CloudSimTags
:   Denotes a request to destroy a new VM in a Datacentre

VM\_DESTROY\_ACK - Static variable in class org.cloudbus.cloudsim.core.CloudSimTags
:   Denotes a request to destroy a new VM in a Datacentre

VM\_MIGRATE - Static variable in class org.cloudbus.cloudsim.core.CloudSimTags
:   Denotes a request to migrate a new VM in a Datacentre

VM\_MIGRATE\_ACK - Static variable in class org.cloudbus.cloudsim.core.CloudSimTags
:   Denotes a request to migrate a new VM in a Datacentre With acknowledgement information sent
    by the Datacentre

VM\_MIPS - Static variable in class org.cloudbus.cloudsim.examples.power.Constants


VM\_PES - Static variable in class org.cloudbus.cloudsim.examples.power.Constants


VM\_RAM - Static variable in class org.cloudbus.cloudsim.examples.power.Constants


VM\_SIZE - Static variable in class org.cloudbus.cloudsim.examples.power.Constants


VM\_STATUS\_BUSY - Static variable in class org.workflowsim.WorkflowSimTags
:   VM Status is busy (no jobs should run on this vm)

VM\_STATUS\_IDLE - Static variable in class org.workflowsim.WorkflowSimTags
:   VM Status is idle (a job can run on this vm)

VM\_STATUS\_READY - Static variable in class org.workflowsim.WorkflowSimTags
:   VM Status is ready (not used)

VM\_TYPES - Static variable in class org.cloudbus.cloudsim.examples.power.Constants


VmAllocationPolicy - Class in org.cloudbus.cloudsim
:   VmAllocationPolicy is an abstract class that represents the provisioning policy of hosts to
    virtual machines in a Datacentre.

VmAllocationPolicy(List<? extends Host>) - Constructor for class org.cloudbus.cloudsim.VmAllocationPolicy
:   Allocates a new VmAllocationPolicy object.

VmAllocationPolicySimple - Class in org.cloudbus.cloudsim
:   VmAllocationPolicySimple is an VmAllocationPolicy that chooses, as the host for a VM, the host
    with less PEs in use.

VmAllocationPolicySimple(List<? extends Host>) - Constructor for class org.cloudbus.cloudsim.VmAllocationPolicySimple
:   Creates the new VmAllocationPolicySimple object.

vmCreate(Vm) - Method in class org.cloudbus.cloudsim.Host
:   Allocates PEs and memory to a new VM in the Host.

vmDeallocate(Vm) - Method in class org.cloudbus.cloudsim.Host
:   Deallocate all hostList for the VM.

vmDeallocateAll() - Method in class org.cloudbus.cloudsim.Host
:   Deallocate all hostList for the VM.

vmDestroy(Vm) - Method in class org.cloudbus.cloudsim.Host
:   Destroys a VM running in the host.

vmDestroyAll() - Method in class org.cloudbus.cloudsim.Host
:   Destroys all VMs running in the host.

vmId - Variable in class org.cloudbus.cloudsim.Cloudlet
:   The vm id.

vmId - Variable in class org.workflowsim.failure.FailureRecord
:   the location.

vmList - Variable in class org.cloudbus.cloudsim.DatacenterBroker
:   The vm list.

vmList - Static variable in class org.cloudbus.cloudsim.examples.power.RunnerAbstract
:   The vm list.

VmList - Class in org.cloudbus.cloudsim.lists
:   VmList is a collection of operations on lists of VMs.

VmList() - Constructor for class org.cloudbus.cloudsim.lists.VmList


Vmlist - Variable in class org.cloudbus.cloudsim.network.datacenter.Switch


vmList - Variable in class org.workflowsim.WorkflowEngine


vmsAcks - Variable in class org.cloudbus.cloudsim.DatacenterBroker
:   The vms acks.

VmScheduler - Class in org.cloudbus.cloudsim
:   VmScheduler is an abstract class that represents the policy used by a VMM to share processing
    power among VMs running in a host.

VmScheduler(List<? extends Pe>) - Constructor for class org.cloudbus.cloudsim.VmScheduler
:   Creates a new HostAllocationPolicy.

VmSchedulerSpaceShared - Class in org.cloudbus.cloudsim
:   VmSchedulerSpaceShared is a VMM allocation policy that allocates one or more Pe to a VM, and
    doesn't allow sharing of PEs.

VmSchedulerSpaceShared(List<? extends Pe>) - Constructor for class org.cloudbus.cloudsim.VmSchedulerSpaceShared
:   Instantiates a new vm scheduler space shared.

VmSchedulerTimeShared - Class in org.cloudbus.cloudsim
:   VmSchedulerTimeShared is a VMM allocation policy that allocates one or more Pe to a VM, and
    allows sharing of PEs by multiple VMs.

VmSchedulerTimeShared(List<? extends Pe>) - Constructor for class org.cloudbus.cloudsim.VmSchedulerTimeShared
:   Instantiates a new vm scheduler time shared.

VmSchedulerTimeSharedOverSubscription - Class in org.cloudbus.cloudsim
:   This is a Time-Shared VM Scheduler, which allows over-subscription.

VmSchedulerTimeSharedOverSubscription(List<? extends Pe>) - Constructor for class org.cloudbus.cloudsim.VmSchedulerTimeSharedOverSubscription
:   Instantiates a new vm scheduler time shared over subscription.

vmsCreatedList - Variable in class org.cloudbus.cloudsim.DatacenterBroker
:   The vms created list.

vmsDestroyed - Variable in class org.cloudbus.cloudsim.DatacenterBroker
:   The vms destroyed.

vmsRequested - Variable in class org.cloudbus.cloudsim.DatacenterBroker
:   The vms requested.

VmStateHistoryEntry - Class in org.cloudbus.cloudsim
:   The Class VmMipsAllocationHistoryEntry.

VmStateHistoryEntry(double, double, double, boolean) - Constructor for class org.cloudbus.cloudsim.VmStateHistoryEntry
:   Instantiates a new vm mips allocation history entry.

vmsToDatacentersMap - Variable in class org.cloudbus.cloudsim.DatacenterBroker
:   The vms to datacenters map.

VmtoHostlist - Variable in class org.cloudbus.cloudsim.network.datacenter.NetworkDatacenter


VmToSwitchid - Variable in class org.cloudbus.cloudsim.network.datacenter.NetworkDatacenter

A B C D E F G H I J L M N O P Q R S T U V W Z

- Overview
- Package
- Class
- Use
- Tree
- Deprecated
- Index
- Help

- Prev Letter
- Next Letter

- Frames
- No Frames

- All Classes
